# Psychometric properties of Fagerström Tolerance Questionnaire among Turkmen Nass (Naswar) users

**DOI:** 10.1186/s13722-023-00390-1

**Published:** 2023-05-23

**Authors:** Bagher Pahlavanzadeh, Shohreh Kolagari, Mohammad Ebrahimi Kalan, Ziyad Ben Taleb, Kenneth D. Ward, Samane Zare, Abdurrahman Charkazi

**Affiliations:** 1 Department of Public Health, Abadan Faculty of Medical Sciences, Abadan, Iran; 2 Faculty of Nursing & Midwifery, Golestan University of Medical Sciences, Gorgan, Iran; 3 School of Health Professions, Eastern Virginia Medical School, Norfolk, VA USA; 4 Department of Kinesiology, College of Nursing and Health Innovation, University of Texas at Arlington, 411 S. Nedderman Drive Box 19407, Arlington, TX 76019-0407 USA; 5 School of Public Health, University of Memphis, 3720 Alumni Ave, Memphis, TX 38152 USA; 6 School of Medicine, Department of Social Medicine, Population, & Public Health, University of California Riverside, 3333 14th Street, Riverside, CA 92501 USA; 7 Environmental Health Research Center, Faculty of Health, Golestan University of Medical Sciences, Late Falsefi University Complex, KM 5of Gorgan-Sari Road, Gorgan, Iran

**Keywords:** Smokeless Tobacco, Nass, Fagerström Tolerance Questionnaire, Turkmen, Iran

## Abstract

**Background:**

Smokeless tobacco (SLT) products are gaining popularity around the globe, particularly in Asia, Africa, and the Middle East. Among these products, Nass (aka Naswar) is popular among the Turkmen ethnicity in Iran. Although several studies reported nicotine dependence (ND) among SLT users, psychometric instruments have never been utilized to specifically measure ND among Nass users. Therefore, in this study, we aimed to evaluate the reliability and validity of the Fagerström Tolerance Questionnaire (FTQ) among Turkmen Nass users.

**Methods:**

A cross-sectional, descriptive study was conducted in June-December 2018 among 411 Turkmen adults who currently (past 30 days) used Nass. Two bilinguals (Persian English) individuals translated and back-translated the FTQ-SLT, which maintained both the questionnaire's accuracy and cultural sensitivity. Construct validity was assessed using exploratory and confirmatory factor analysis.

**Results:**

The mean age and standard deviation for initiating Nass were 22.5 ± 11.81 years. Exploratory and confirmatory factor analysis indicated a single-factor solution with 8-items that captured several important ND components. Using Nass frequently, soon after waking, when sick, and experiencing a craving were some of the main components. Subgroups comparison revealed that higher scores occurred among those who were married, had Nass user(s) in their immediate family, and consumed bulk form of Turkmen Nass directly without using a tissue.

**Conclusion:**

Our findings show that the FTQ- SLT is a fairly reliable and valid scale to measure ND among Turkmen Nass users and warrants further testing to accommodate cross-cultural differences in other populations.

## Introduction

Tobacco use causes more than 8 million deaths every year worldwide [1]. Although most tobacco-related morbidity and mortality are linked to combustible tobacco products, using non-combustible forms of tobacco such as smokeless tobacco (SLT) exposes users to cancer-causing chemicals and can lead to nicotine dependence (ND) and dual use of SLT and combustible Tobacco [2–6]. Nass (aka Nasvai, Naswār) is a form of SLT popular in South and Central Asian countries such as Iran, Pakistan, Turkmenistan, Afghanistan, Kyrgyzstan, Uzbekistan, Tajikistan, and India [6–8]. This version of SLT contains a combination of tobacco leaves, lime, cotton oil or sesame oil, and ash with the optional addition of condiments like green cardamom, menthol, and indigo [9, 10]. Although in Kazakhstan, Nass was added to the list of narcotic and psychoactive substances in 2011 [11], other parts of the world––especially the aforementioned countries––are enjoying a relaxed regulatory environment for this addictive substance.

In Iran, Nass use is popular among males of Turkmen ethnicity [8, 12]. Typically, Nass users keep the product between their gums and lower lip for about 2–10 min before spitting it out [12]. The Nass used by Turkmen comes in 50 g plastic packages. Unlike many types of SLT, especially in some Western countries that are pasteurized during the manufacturing process to kill bacteria that can produce cancer-causing chemicals and bacterial infections [13], Nass in developing countries (e.g., Iran) contains fire-cured and fermented tobacco [14]. A cohort study of 50,000 individuals from Iran found that current Nass use was associated with overall mortality (Hazard Ratio = 1.16; 95% CI 1.01–1.34); there was  > 60% higher risk of cancer death in people using Nass more than five times a day [15]. This fact is concerning and calls for further investigation into this type of SLT to curb associated morbidity and mortality in this region.

Turkmen and Afghan Nass are two popular types of Nass in Iran [12]. Turkmen Nass is a mixture of ground tobacco with ash, salt, and water; older men are usually the primary consumers of it [12]. While, Afghan Nass is more common among young people and is usually made with lime, salt, water, pepper, and other synthetic materials [12].

Like other tobacco products, SLT products contain nicotine and can cause ND, evidenced by craving and other withdrawal symptoms following short abstinence [16] and compulsion to use despite the harmful effects [6, 16]. Many users are unable to quit SLT due to the formidable grip of nicotine. Several studies have developed psychometrically sound tools to assess ND among SLT users [17]. In 1995, Boyle et al., introduced two modified versions of the Fagerström Tolerance Questionnaire (FTQ) to assess ND in SLT users [18]. The FTQ requires an SLT brand nicotine content rating, but variability exists in the nicotine content between batches of the SLT products in Iran [8] and beyond [19] To dodge the need to assess SLT brand nicotine content, which is a huge challenge due to this variability, the Fagerström Test for Nicotine Dependence (FTND) was adopted [20]. In fact, the earlier scales to measure SLT-specific ND applied the modified FTQ and the FTND, which resulted in the Fagerstrum Smokeless Tolerance Questionnaire (FTQ-SLT) that can predict serum cotinine and the frequency of SLT use [18, 21].

An important challenge that intercultural research faces is that most of the SLT-specific ND-measuring tools are developed in English-speaking countries with relatively few tools properly standardized in non-English-speaking nations––particularly in low- and middle-income countries. Due to differences in cultural cues to use tobacco products [22, 23] and interpretation of items, it is not guaranteed that psychometric integrity (validity and reliability) will remain intact following the translation of the tool into another language. Therefore, it is crucial to assess the psychometric properties of the instrument and modify it as needed based on cultural differences prior to employment [24]. Additionally, differences in determinants of initiating and continuing use, as well as different ingredients or formulations may impact how ND is experienced. Also, based on our previous qualitative study [12] among Turkmen Nass users, we hypothesize that using Nass directly without tissue paper (bulk form) can be an indicator of immediate consumption due to craving or urge to use Nass at the moment compared to those who are using tissue. To the best of our knowledge, there is no validated scale measuring SLT-specific ND in Iran. Therefore, in this study, we aimed to determine the psychometric properties of the FTQ-SLT among Turkmen adults using Nass in Golestan province in the northeast of Iran.

## Methods

### Study design and sample

This cross-sectional study comprises 411 adult current Nass users who were selected using a convenience sampling method. Study flyers were distributed in cities and villages of the studied province (i.e., AqQala, Bandar-e-Turkmen, and Gomishan). Participants were invited to part take in the study if they met inclusion criteria including being at least 18 years of age, used Nass at least once in the past 30 days and at least one pack per week in the past year, and did not report psychological problems such as depression. The Institutional Review Board of Golestan University of Medical Sciences approved the study protocol (IR.GOUMS.REC.13943248).

### Measures

#### Demographic and Nass use patterns

This questionnaire included age, marital status (single/married), occupation (unemployed, self-employed, employed, and student), education (Illiterate, primary and secondary, high school, and University degree), self-declared SES (as Poor, Average, Good), number of times used Nass per day, type of Nass use (Turkmen/Afghan), number of friends who use Nass, having brother(s)/father or both who use Nass, using other tobacco products/substances (cigarettes, hookah, Opium, At least one of the crack cocaine, crystal, or cannabis), the longest period of quitting Nass (months), and age at initiating Nass use.

#### Fagerstrom Tolerance Questionnaire for smokeless tobacco (FTQ-SLT)

The FTQ-SLT is a 10-item ND scale, with a range of possible scores between 4 and 19; a higher score indicates greater ND. Previous studies showed that FTQ-SLT was significantly correlated with baseline saliva cotinine in SLT users; ranging from r = 0.17 to r = 0.70. [17, 18, 25] Because Nass users are not aware of its nicotine content and do not swallow the tobacco juices [12], we removed two items (i.e., “nicotine content and how often do you swallow tobacco juices”), leaving 8 items.

In the first step, the 8-item FTQ-SLT was translated into Persian by the authors, and then this translated version was given to two bilinguals (Persian-English) who back-translated it into English without access to the original version. The translation was matched with the original version and its content structure, and after some wording changes to clarify meaning, it was finalized. Next, we conducted a pilot study with 50 Nass users to better understand whether participants understood the meaning of items and response choices and to estimate the time required to complete the instrument.

### Data analysis

In the first step, construct validity of the Persian version of FTQ-SLT was assessed by Exploratory Factor Analysis (EFA) with varimax rotation on a randomly selected subsample of subjects (55% of total subjects). In this analysis, the adequacy of the sample and the sphericity of the covariance matrix were assessed through the Kaier-Meyer-Olkin (KMO) criterion and the Bartlett test [26, 27]. The number of factors with an eigenvalue greater than 1 was used to determine the number of factors. Next, a Confirmatory Factor Analysis (CFA) was carried out to assess the appropriateness of the proposed model in EFA on another subsample (45% of total subjects). In line with literature [28] that suggests using the separate samples for EFA and CFA as a desirable approach, we used a random split approach to achieve random samples for both psychometric measures (i.e., EFA and CFA). We used SPSS to split data randomly into two equal subsets (~ 50%). SPSS yielded two subsets with n = 219 subjects for EFA and n = 185 for CFA. Due to the categorical measurement scale of items, the diagonally weighted least squares (WLSMV) estimation method was adopted [29]. Goodness of fit of the proposed model was assessed using the root mean square error of approximation (RMSEA), comparative fit index (CFI), Tucker–Lewis index (TLI), and weighted root mean square residual (WRMR) indices. CFI, values close to or higher than 0.90 suggest an adequate fit; TLI, values close to 1.00 are satisfactory, but those between 0.80 and 0.90 are also acceptable; Standardized Root Mean Square Residual (SRMR), values less than 0.5 are considered satisfactory, however values close to 0.8 may also be acceptable; and RMSEA, values between 0.05 and 0.08 are recommended, accepting those up to 0.1. [30, 31]

Finally, to evaluate the external validity, the average factor score was compared between subgroups of demographic variables using ANOVA, two-sample t-test, Kruskal–Wallis, and Mann–Whitney tests. The EFA was performed using SPSS 21 software and CFA was performed using Mplus7.2 software at a significant level of α = 0.05. The reliability of the instrument was evaluated using the internal consistency method (Cronbach's alpha coefficient).

## Results

### Descriptive characteristics

Table 1 shows that of the 411 participants, the mean and standard deviation (SD) of age was 38.8 ± 14.8 years with a range of 16–85 years, 78.3% were unmarried, 50.1% were middle class, and 96.1% had high school or less than high school education. The mean (SD) age of Nass use initiation was 22.5 ± 11.2 years. Most participants (84.4%) reported that their fathers or siblings used Nass. Afghan Nass (90%) was the most popular type of Nass, and most participants (76.4%) did not use clean tissue paper when they consumed it. Table 2 shows that more than 71% of participants stated that if they do not use Nass for about 2 h, their craving/desire will increase. Most participants (65.2%) also reported that they consume Nass during the first 30 min after waking up. Only 27.7% of participants always keep Nass in their mouth.

**Table 1:** Sociodemographic characteristics and tobacco/substances use pattern of the study population (N = 411)

Variable	n/mean	%/SD
Age (mean/SD)	38.8	14.8
Age first used Nass (mean/SD)	22.5	11.2
Self-declared SES*		
Poor	159	38.7
Average	206	50.1
Good	46	11.2
Education		
Illiterate	28	6.8
Primary and secondary	260	63.3
High school	107	26
University graduate	16	3.9
Occupation e		
Unemployed	88	21.4
Self- employed	300	73
Employe	17	4.1
Pupil	6	1.5
Type of Nass		
Afghan Nass	370	90
Turkmen Nass	38	9.2
Both	3	0.8
Form of use		
Bulk form	314	76.4
Wrapped in tissues	67	16.3
Both	30	7.3
Nass use among family members		
Father	171	41.6
Brother(s)	138	33.6
Both (brother(s) and father)	64	15.6
Other types of substance use		
Cigarettes		
Current smoker	117	28.5
Never smoker	225	62
Quit	39	9.5
Hookah		
Current smoker	31	7.5
Never smoker	380	92.5
Opium		
Current user	109	26.5
Never user	302	73.5
Ever use of crack cocaine, crystal meth or cannabis		
Current smoker	27	6.6
Never smoker	384	93.4

*SES, Socioeconomic status was measured based on self-declared information as poor, average, and high

**Table 2:** Status of response to smoke dependence questionnaire items and factor loads on each of the two factors in exploratory factor analysis

Item	Answers	N (%)	Loading factors	
			1st factor	2nd factor
1. After a normal sleeping period, do you use smokeless tobacco within 30 min of waking?	No Yes	143 (34.8) 268 (65.2)	0.749	− 0.145
2. Is it difficult for you not to use smokeless tobacco where its use would be unsuitable or restricted?	No yes	148 (36) 263(64)	0.555	0.279
3. Do you use smokeless tobacco when you are sick or have mouth sores?	No yes	191 (46.5) 220 (53.5)	0.577	− 0.315
4. Do you keep a dip or chew in your mouth almost all the time?	No yes	297 (72.3) 114 (27.7)	0.484	0.131
5. Do you experience strong cravings for a dip/chew when you go for more than 2 h without one?	No yes	118 (28.7) 293 (71.3)	0.594	0.036
6. On average, how many minutes do you keep a fresh dip or chew in your mouth?	< 20 20 to 30 > 30	373 (90.8) 26 (6.3) 12 (2.9)	0.010	0.922
7. On average, how many dips or chews do you take each day?	1–9 10–20 > 20	166 (40.4) 106 (25.8) 139 (33.8)	0.787	0.064
8. How many days does a tin/can last you?	6–7 3–5 < 3	53 (12.9) 149 (36.3) 209 (50.9)	0.721	− 0.189

### Exploratory factor analysis (EFA)

In this study, EFA was performed for 219 sample units. The principal component method was used to extract the factors, and the varimax method was used to rotate the factor loads. KMO test showed the adequacy of the number of studied samples (KMO = 0.813). The Bartlett test also showed the appropriateness of factor analysis for these data χ^2^ = 329, df = 28, *P* value < 0.001.

Using an eigenvalue of > 1 as a cut-off, two factors emerged; The first factor explained 36.8% of the variance, and the two-factor model explained 50.6%. The second factor contained only one item (item 6: “On average how many minutes do you keep a fresh dip or chew in your mouth?”).* This item loaded* very poorly on the first factor (0.010) and had little variability in responses (more than 90% of respondents reported < 20 min). Therefore, the 6-item, a one-factor solution was used, for which factor loadings ranged from 0.484 to 0.787 (Table 2), and internal consistency was adequate (α = 0.645). The model obtained from this step was examined in Confirmatory Factor Analysis (CFA; see below).

### Confirmatory factor analysis (CFA)

**Fig. 1:**
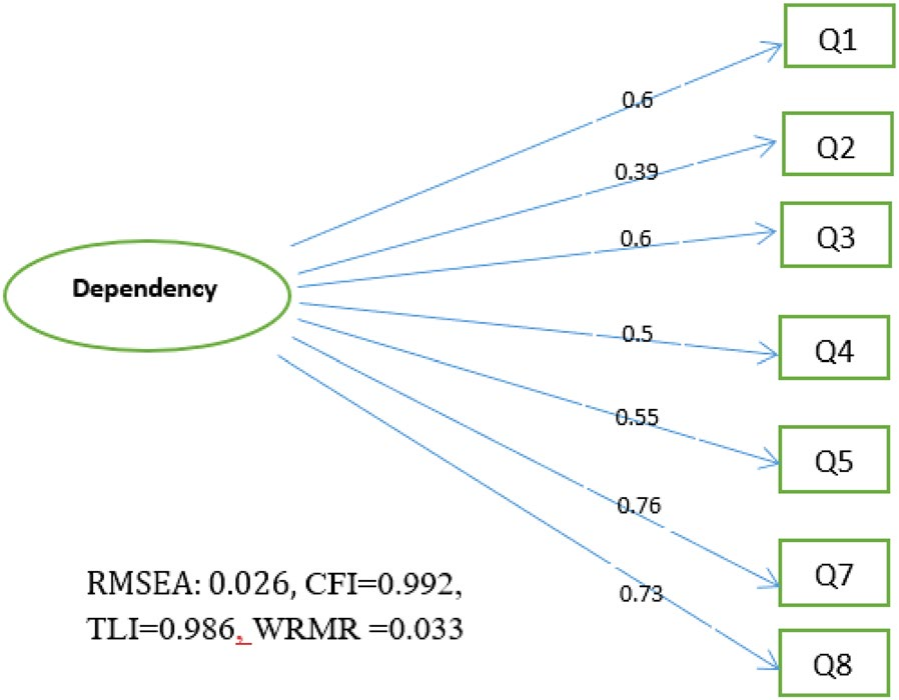
Confirmatory factor analysis of FTQ-SLT among Turkmen Nass users (n = 185)

As shown in Fig. 1, the CFA for the one-factor model was performed on the data of 185 subjects. All indices indicated good model fit, including the chi-square test $(150.6,\mathrm{p}<0.001)$, RMSEA indicator (95% CI): 0.026 (0.001, 0.091), CFI = 0.992, TLI = 0.986 and WRMR indicator = 0.033.

Standardized factor loadings from the CFA of FTQ-SLT among Turkmen Nass users are presented in Fig. 1. These loadings indicate that ND in the Iranian Turkmen male population is mainly explained by items 1, 7, and 8, with item 2 having the least explanatory power among items. In other words, the higher the “average number of daily doses”, the shorter the “time it takes to consume a can” and using Nass within 30 min of waking are indicative of greater ND.

### External validity

Table 3 indicates a comparison of FTQ-SLT based on sociodemographic. The standardized mean factor score was significantly lower for unmarried individuals compared to their married counterparts (p = 0.032). ND was greatest among respondents who had Nass users in their immediate family (both father and brother(s)) (p = 0.004). The mean standardized factor scores were greater among those who used Nass without tissue compared to those who sometimes or always used tissue p = 0.003); scores did not differ significantly according to SES and whether respondents ever smoked cigarettes, hookah, opium, or other substances compared to people who consumed Nass only.

**Table 3:** Comparison of the mean of dependence on smokeless tobacco in people consuming Nass in Iranian people

Variable	Groups	N (%)	Tolerance^§^ Mean (SD)	P-value
Marital Status	Single Married	89 (21.7) 322 (78.3)	− 0.14 (0.6) 0.02 (0.63)	0.032
SES	Weak Moderate Good	159 (38.7) 206 (50.1) 46 (11.2)	0.06 (0.63) − 0.05 (0.62) − 0.14 (0.63)	0.074
Nass use among family members	No Father or Brother Both	166 (40.4) 181 (44) 64 (15.6)	− 0.11 (0.62) − 0.001 (0.63) 0.2 (0.58)	0.004
Nass type	Afghani Turkmen	370 (90) 38 (9.2)	− 0.001 (0.63) 0.32 (0.55)	0.16
Form of use	Bulk form Wrapped in tissue Both	314(76.4) 67(16.3) 30(7.3)	0.035(0.61) − 0.24(0.65) 0.00(0.69)	0.003
Cigarette	Never smoker Current smoker Quit	255(62) 117(28.5) 39(9.5)	− 0.01(0.63) − 0.02(0.64) 0.01	0.9
Hookah	Never smoker Current smoker	380(92.5) 31(7.5)	− 0.03 − 0.15	0.2
Opium	Never user Current user	302(73.5) 109(26.5)	− 0.05 0.08	0.1
Ever use of crack cocaine, crystal meth, or cannabis	Never smoker Current smoker	384(93.4) 27(6.6)	− 0.013(0.64) − 0.018(0.5)	0.86

^§^the standardized mean of factor score

## Discussion

In this study, we adapted the FTQ-SLT for a previously unstudied tobacco product (Nass) and psychometrically evaluated a 8-item version of this instrument with Turkmen consumers living in the Golestan province of Iran. EFA and CFA determined that the 8-items loaded on a single factor reflecting several established characteristics of tobacco dependence [32, 33], including frequent and early morning use, craving, and continued use even when sick.

The low factor loading of item number 2 (difficulty not smoking where use would be unsuitable or restricted) can be explained by the fact that no developed and implemented regulations prohibit using Nass in public places in Iran. Also, Nass use can be easily disguised. For example, suppose Nass use is discouraged at a party or funeral; In that case, the user can go to a secluded place and put a piece of paper tissue under the tongue, which is less conspicuous. The inconspicuousness of Nass may make enforcement of tobacco control regulations challenging.

Our results are consistent with those of Ebbert and colleagues. They found that FTQ-SLT scores were positively associated with the intensity of use (the number of cans of SLT consumed per week) and early morning use of SLT, which is consistent with patterns of ND for other tobacco products [34, 35].These researchers also showed that high scores on the FTQ-SLT were correlated with greater serum nicotine and cotinine levels [35] Unfortunately, we could not assess nicotine exposure in our study and future studies will need to evaluate this association in Nass users.

The reliability (internal consistency) of the FTQ-SLT for Nass users was moderate and in line with the instrument's reliability for other groups of SLT users. For example, Ebbert et al. [20] and Ferketich et al. [33] reported Cronbach's alpha of 0.47 and 0.40, respectively, which are lower than what we found in our study (0.645). The number of questions used to measure FTQ-SLT could affect the reliability of the instrument. For instance, a previous similar study used 6-item FTQ-SLT and reported reliability of 0.72, which was higher than this study and others [36]. As ours is the first psychometric evaluation of the FTQ-SLT for Nass users, future research should assess its reliability across different populations and determine whether the existing instrument does not adequately capture some Nass use features that may relate to ND. For example, while an item assessing difficulty refraining from Nass use where it’s restricted may not be useful due to lack of tobacco control regulations, a more germane item might capture the frequency of using tissue paper to disguise consumption, which might reflect ND processes such as greater behavioral priority or loss of control.

This study has limitations that should be considered in generalizing the results. First, this study’s serum cotinine levels were not obtained due to lack of access to related kits and budget constraints. In most studies in Western societies [35, 36], self-report ND instruments have been assessed against biochemical measurements such as serum or saliva cotinine, and the same needs to be done among Nass users. Although Iran is still under economic restrictions, we hope to obtain funding to evaluate serum or saliva cotinine to investigate the dependency rate in a larger sample of Turkmen Nass users in future studies. This is crucial since this ethnicity is at great risk of initiating and continuing use of Nass and developing ND, which may lead to up taking other combustible tobacco products, further increasing the risk of premature morbidity and mortality [15]. Another limitation is not including females in our sample due to small rate of Nass use among females in the Turkmen population. Also, the self-declared measure of SES may not capture the whole picture of the SES and awaits future studies to investigate this important factor among Turkmen Nass users. Lastly, it is important to establish the FTQ-SLT’s predictive validity in this population against key outcomes such as difficulty quitting and escalating use. Despite these limitations, this study provides insight into ND among Nass users of Turkmen ethnicity who are prone to initiate this tobacco use mode earlier in their life. Future studies may look at the trajectories of ND among Nass-only and dual users of Nass and combustible/non-combustible tobacco products. Although we did not report content validity index (CVI) or content validity ratio (CVR), to capture some level of content validity for the developed scale for Turkmen population, we removed 2 items from the original scales: (i) Nicotine content: Nass in the Golestan province are prepared in the bulk form without clear nicotine contents and (ii) consumption mode: it is normally consumed by putting in sublingual form and not swallowed. Nevertheless, future studies should consider CVI and CVR in order to increase the validity of scales in this population or others.

## Conclusion

This study gives preliminary evidence of the utility of an adapted version of the FTQ-SLT to assess ND among Iranian Turkmen Nass users. Improving our understanding of ND processes for this popular and understudied form of tobacco use is needed to shed light on epidemiological and clinical studies and develop culturally sensitive cessation interventions.

## Data Availability

Data will be available at reasonable request from corresponding author via E-mail.

## Abbreviations

SLT: Smokeless tobacco
ND: Nicotine dependence
FTQ: Fagerström Tolerance Questionnaire
FTND: Fagerstrom test for nicotine dependence
FTQ-SLT: Fagerstrum smokeless tolerance questionnaire
EFA: Exploratory factor analysis
KMO: Kaier–Meyer–Olkin
CFA: Confirmatory factor analysis
WLSMV: Weighted least squares
RMSEA: Root mean square error of approximation
CFI: Comparative fit index
TLI: Tucker–Lewis index
WRMR: Weighted root mean square residual
SRMR: Standardized root mean square residual
SD: Standard deviation
CVI: Content validity index
CVR: Content validity ratio
